# Insights into the biocontrol and plant growth promotion functions of *Bacillus altitudinis* strain KRS010 against *Verticillium dahliae*

**DOI:** 10.1186/s12915-024-01913-1

**Published:** 2024-05-20

**Authors:** Yujia Shan, Dan Wang, Fu-Hua Zhao, Jian Song, He Zhu, Yue Li, Xiao-Jun Zhang, Xiao-Feng Dai, Dongfei Han, Jie-Yin Chen

**Affiliations:** 1 The State Key Laboratory for Biology of Plant Diseases and Insect Pests, Institute of Plant Protection, Chinese Academy of Agricultural Sciences, Beijing, 100193 China; 2 College of Life Science and Technology, Mudanjiang Normal University, Mudanjiang, 157012 China; 3 State Key Laboratory of Subtropical Silviculture, School of Forestry and Biotechnology, Zhejiang A&F University, Hangzhou, 311300 China; 4 The Cotton Research Center of Liaoning Academy of Agricultural Sciences, National Cotton Industry Technology System Liaohe Comprehensive Experimental Station, Liaoning Provincial Institute of Economic Crops, Liaoyang, 111000 China; 5 Western Agricultural Research Center, Chinese Academy of Agricultural Sciences, Changji, 831100 China; 6 School of Environmental Science and Engineering, Suzhou University of Science and Technology, Suzhou, 215009 China; 7 State Key Laboratory of Efficient Utilization of Arid and Semi-arid Arable Land in Northern China, Beijing, 100081 China

**Keywords:** *Bacillus altitudinis*, *Verticillium dahliae*, Biocontrol, Plant immunity, Plant growth promotion

## Abstract

**Background:**

Verticillium wilt, caused by the fungus *Verticillium dahliae*, is a soil-borne vascular fungal disease, which has caused great losses to cotton yield and quality worldwide. The strain KRS010 was isolated from the seed of Verticillium wilt-resistant *Gossypium hirsutum* cultivar “Zhongzhimian No. 2.”

**Results:**

The strain KRS010 has a broad-spectrum antifungal activity to various pathogenic fungi as *Verticillium dahliae*, *Botrytis cinerea*, *Fusarium* spp., *Colletotrichum* spp., and *Magnaporthe oryzae*, of which the inhibition rate of *V. dahliae* mycelial growth was 73.97% and 84.39% respectively through confrontation test and volatile organic compounds (VOCs) treatments. The strain was identified as *Bacillus altitudinis* by phylogenetic analysis based on complete genome sequences, and the strain physio-biochemical characteristics were detected, including growth-promoting ability and active enzymes. Moreover, the control efficiency of KRS010 against Verticillium wilt of cotton was 93.59%. After treatment with KRS010 culture, the biomass of *V. dahliae* was reduced. The biomass of *V. dahliae* in the control group (Vd991 alone) was 30.76-folds higher than that in the treatment group (KRS010+Vd991). From a molecular biological aspect, KRS010 could trigger plant immunity by inducing systemic resistance (ISR) activated by salicylic acid (SA) and jasmonic acid (JA) signaling pathways. Its extracellular metabolites and VOCs inhibited the melanin biosynthesis of *V. dahliae*. In addition, KRS010 had been characterized as the ability to promote plant growth.

**Conclusions:**

This study indicated that *B. altitudinis* KRS010 is a beneficial microbe with a potential for controlling Verticillium wilt of cotton, as well as promoting plant growth.

**Supplementary Information:**

The online version contains supplementary material available at 10.1186/s12915-024-01913-1.

## Background

Cotton belongs to the genus *Gossypium* in the family Malvaceae and produces the most important textile fiber, as it contributes about 35% of total nature fiber for the textile industry [1], and also serves as one of the sources of edible oil and livestock feed [2]. Among them, upland cotton *Gossypium hirsutum* is the most widespread and encompasses 95% of global cotton production [3]. Throughout their life span, cotton plants confront an endless barrage of pathogens and pests, of which Verticillium wilt, caused by *Verticillium dahliae*, is known as a vascular soil-borne disease, which has a great effect on the yield and quality of cotton [4]. The dormant structure microsclerotium of *V. dahliae* can survive for up to 14 years in the absence of a host or under adverse conditions [5] and plays a key role in the disease cycle of Verticillium wilt of crops [6], which is one of the important reasons why Verticillium wilt is difficult to control.

In recent years, plant Verticillium wilt has become increasingly serious due to climatic variation, long-term monoculture, and frequent introduction of new cotton varieties/hybrids in various countries and regions in the world [7]. In addition, due to the stable dormant structure microsclerotia, long-term variability, and coevolution with host plants, it is still challenging to control the spread of *V. dahliae* [8]. Common control measures for Verticillium wilt include breeding of resistant cultivars, change of cropping pattern, and chemical control. However, breeding resistant cultivars is limited by the lack of germplasm resources with immunity or high resistance against *V. dahliae*. The cycle of prevention and control for soil-borne disease is long after the change of cropping pattern [9]. Chemical fungicides have been the most widely used method of managing phytopathogens with recent annual use topping one million tons in China. However, the control spectrum of new chemical pesticides is insufficient, and there are still some potential environmental side effects [10]. Continued climate change and agricultural pollution are spurring the creation of environmentally friendly agricultural products, such as safe plant protection solutions [11]. For the past few years, biological control based on functional microorganisms has become a focus of attention, which would satisfy the requirements of sustainable agriculture for environmentally friendly disease control. Biological management with beneficial bacterial strains is one of the environmentally acceptable approaches for lowering plant phytopathogens [12].

Currently, biocontrol bacteria, including the species from *Bacillus*, *Pseudomonas*, and other genera involved in plant growth promotion-related bacteria used in the prevention and management of plant diseases [13], are widely known for their antagonistic activity and diverse beneficial effects on plant health [14]. Their biocontrol mechanisms include the production of antimicrobial substances, competition for ecological niches or substrates, production of inhibitory allelochemicals, and the induction of systemic resistance (ISR) [15]. These bacteria are among the greatest possibilities for the development of biocontrol since they have advantageous effects on a wide variety of plants. Despite these positive characteristics, biocontrol bacteria still show some inconsistency between trials, which is probably due to the short persistence of bacterial cells in the rhizosphere or soil and their susceptibility to unfavorable environmental conditions. One possible way to overcome these drawbacks is to develop inoculants based on beneficial endophytic bacteria [16]. *Bacillus* is one of the most studied genera and has been shown to induce plant resistance [17], promote plant growth [18], and produce antagonistic substances [19]. For example, the fermentation broth of *Bacillus altitudinis* JSCX-1 could inhibit the mycelial growth and zoospore germination of *Phytophthora sojae*, and the biocontrol efficiency of JSCX-1 against *P. sojae* was up to 49.28% in greenhouse experiments [20]. In the detached leaves and potted plant assays, *Bacillus amyloliquefaciens* DB2 had remarkable inhibition activity against *Bipolaris sorokiniana*, and the control efficacy was up to 75.22%. Furthermore, the fermentation broth of DB2 had a significant promotion for wheat seedling growth [21].

The most effective environmental technique for preventing Verticillium wilt in cotton is biological management [22]. This technique is ideal for preventing the growth of fungi and lowering pesticide residue levels while cultivating crops [23]. The metabolites extracted from microorganisms can also be used as an ideal substitute for chemically synthesized antifungal agents to improve the quality of agricultural products [24]. *Bacillus velezensis* XT1 reduced Verticillium incidence rate and percentage of severity by 54 and 80%, respectively [25]. *Bacillus subtilis* EBS03 fermentation broth root irrigation had the highest controlling effect at 87.11% on cotton Verticillium wilt [26]. These biocontrol bacteria’s metabolites will be a significant source of antibiotic compounds to prevent Verticillium wilt. *B. altitudinis*, in particular, is considered probiotics that have no harmful effects on the environment or the human being [27]. As a result, *B. altitudinis*, which can effectively antagonize *V. dahliae*, is a potential biocontrol strain for controlling Verticillium wilt in cotton.

In this study, the strain KRS010 was isolated from the seed of Verticillium wilt-resistant *Gossypium hirsutum* cultivar “Zhongzhimian No. 2.” To better understand this strain, the broad-spectrum antifungal activity was evaluated against a variety of pathogenic fungi. Following that, a series of experiments were performed, and the classification of KRS010 was determined by morphological, physiological, and biochemical characteristics and phylogenetic analysis. We studied the effects of KRS010 on the growth and development of the mycelium and the potential of melanin synthesis in *V*. *dahliae* and evaluate the effects of KRS010 culture filtrate on the pathogen control and growth promotion for cotton as well. This work will provide an effective biological agent to control Verticillium wilt and promote cotton growth.

## Results

### The strain KRS010 has a broad-spectrum antifungal activity

**Fig. 1:**
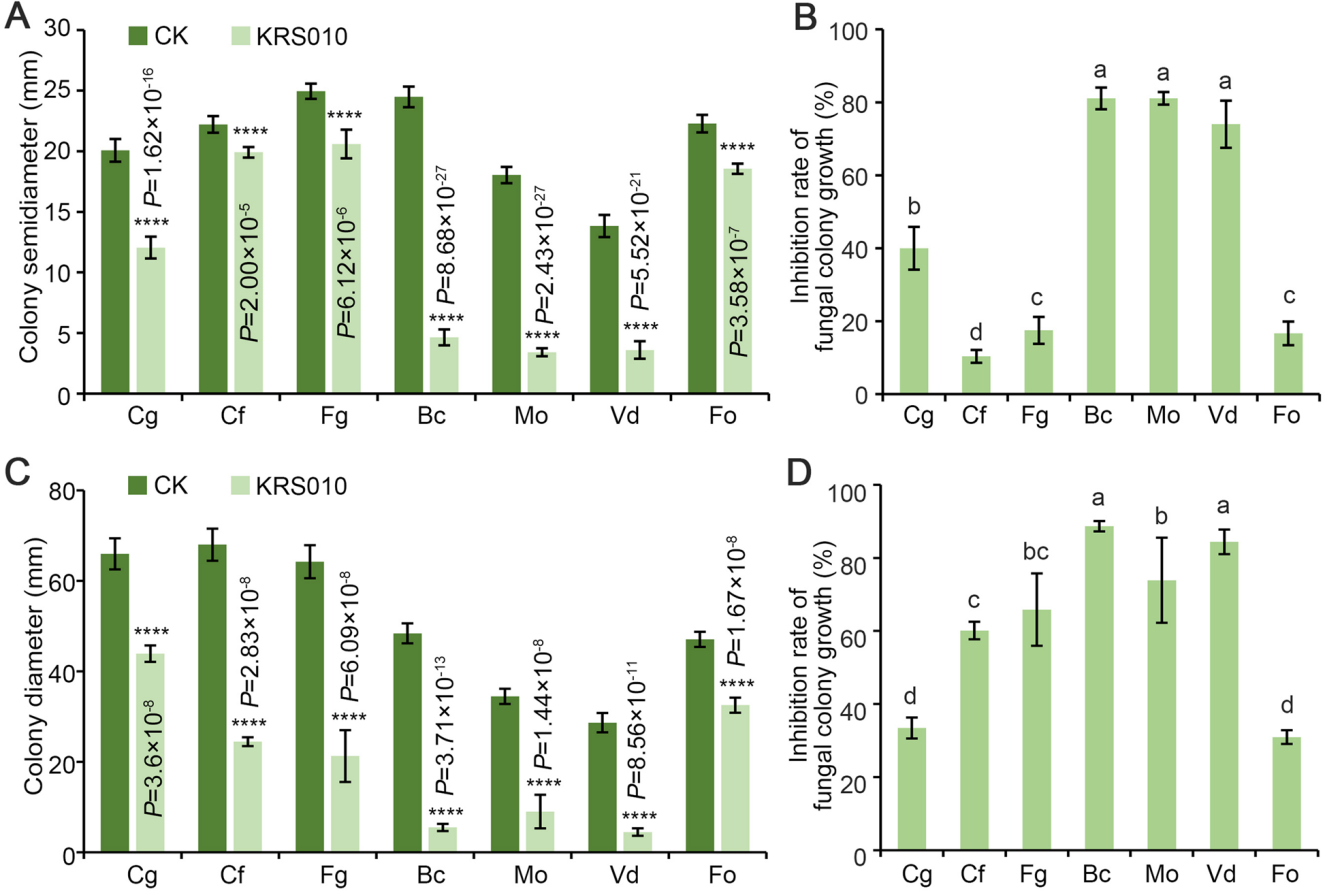
The broad-spectrum inhibition activity of strain KRS010 against phytopathogenic fungi. **A** The semidiameter of the fungal colony by confrontation culture assay. **B** The inhibition rate of fungal colony growth using confrontation culture. **C** The diameter of the fungal colony by the covering fumigation. **D** The inhibition rate of fungal colony growth using the covering fumigation. Inhibition rate calculation: each treatment/group in every experiment was conducted on three plates. Error bars represent standard errors. ****Significant differences at *P* < 0.0001 according to the unpaired Student’s *t* test. The letters a to d above the columns represent the significant differences at *P* < 0.01 according to one-way analysis of variance (ANOVA) and least significant difference (LSD). Cg, *Colletotrichum gloeosporioides*; Cf, *Colletotrichum falcatum*; Fg, *Fusarium graminearum*; Bc, *Botrytis cinerea*; Mo, *Magnaporthe oryzae*; Vd, *Verticillium dahliae*; Fo, *Fusarium oxysporum*; KRS010, KRS010 fermentation broth

In order to test the inhibitory activity of KRS010 against pathogenic fungi, a confrontation test and the volatile organic compound (VOC) treatment were conducted. In the confrontation test, strain KRS010 showed a strong inhibitory effect on the mycelial growth of seven pathogens (Fig. 1A; Additional file 2: Fig. S1A), of which the inhibitory rate of KRS010 against *M. oryzae*, *B. cinerea*, and *V. dahliae* was up to 81.08%, 81.06%, and 73.97%, respectively (Fig. 1B), and this inhibitory rate was 39.97%, 42.31%, 35.60%, and 43.52% for *C. gloeosporioides*, *C. falcatum*, *F. graminearum*, and *F. oxysporum*, respectively (Fig. 1B). The antifungal activity of VOCs released from KRS010 was also detected by covering fumigation assay, in which KRS010 exhibited an inhibitory effect on seven pathogenic fungi at different degrees (Fig. 1C; Additional file 2: Fig. S1B). The colony growth of *V. dahliae* and *B. cinerea* was inhibited with an inhibitory rate above 80% (Fig. 1D). In addition, the inhibition rates of KRS010 VOCs against *C. gloeosporioides*, *C. falcatum*, *F. graminearum*, *M. oryzae*, and *F. oxysporum* were 33.44%, 60.13%, 65.85%, 73.89%, and 30.96%, respectively (Fig. 1D). Together, the detection for antifungal activity suggested that the strain KRS010 exerted a broad-spectrum inhibitory effect on plant pathogens by secreting antagonistic metabolites and releasing VOCs.

### The strain KRS010 was identified as B. altitudinis

**Fig. 2:**
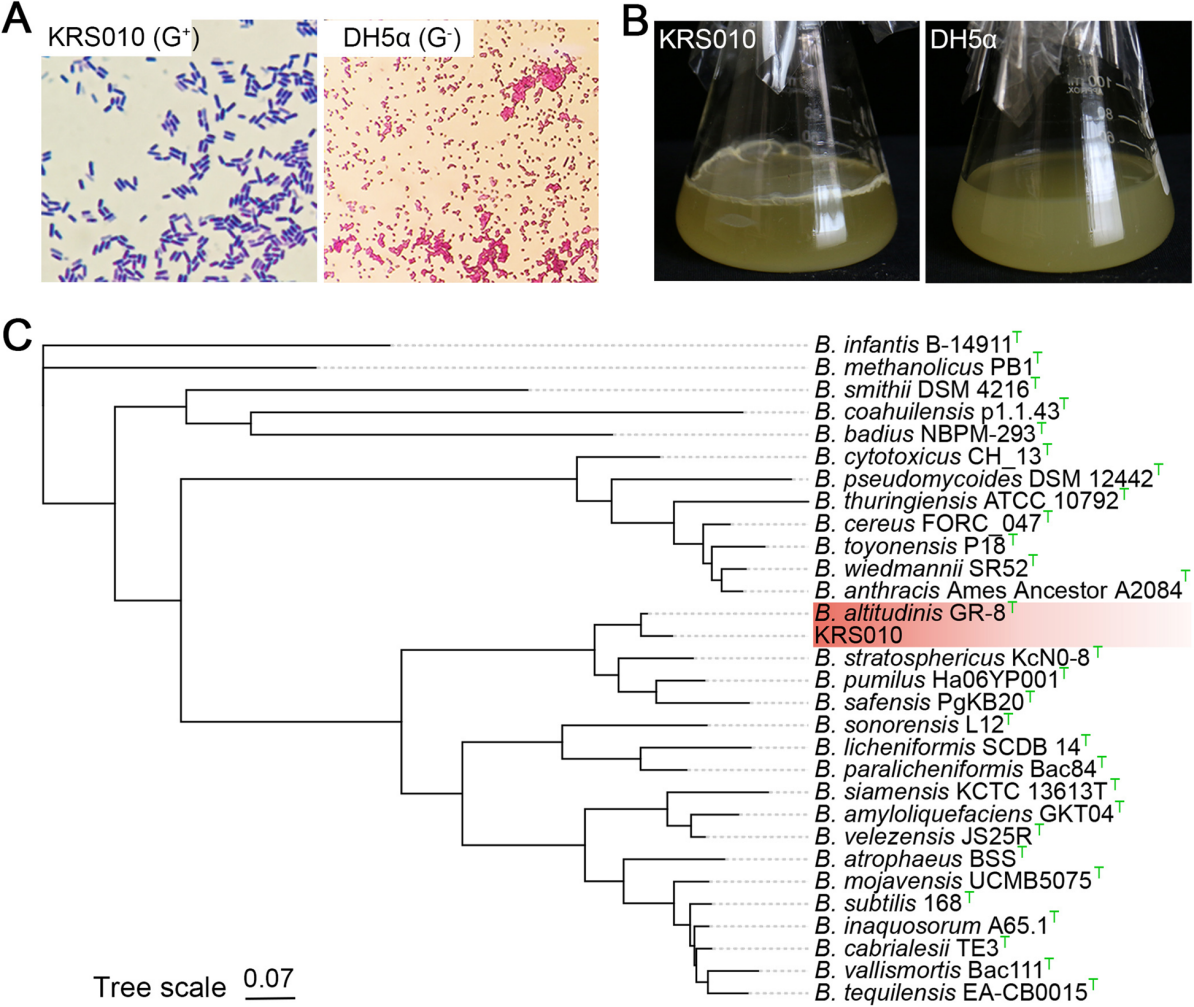
Morphological and molecular identification of KRS010. **A** Gram strain of *E. coli* DH5α served as negative, and *Bacillus altitudini*s KRS010 is positive. **B** Biofilm phenotype of strain KRS010. *E. coli* DH5α served as negative. **C** Maximum likelihood phylogenetic analysis based on complete genome sequences of KRS010 and type strains of other *Bacillus* species. The phylogenetic reconstruction was performed with PhyloPhlAn 3.0.2. The “T” in the superscript of the strain name stands for the type strains of different *Bacillus* species

The taxonomy of strain KRS010 was identified by morphological observation and phylogenetic analysis. The single colony of KRS010 is round or oval, with smooth-faced, easy to pick, milky white, transparent and moist, after incubation for 24 h on LB plate (Additional file 2: Fig. S2A). Isolate KRS010 was identified as gram-positive and bacilliform bacterium by Gram-staining (Fig. 2A). Moreover, KRS010 could form the biofilms at the surface of the static LB liquid medium, the *E. coli* DH5α was served as negative control (Fig. 2B). Scanning electron microscope (SEM) observation indicated that KRS010 is a rod-shaped bacterium, and the ranges of length and width are 1.5 to 2.5 μm and 0.5 to 0.8 μm, respectively (Additional file 2: Fig. S2B). A phylogenetic analysis of KRS010 and type strains of other *Bacillus* species were conducted with Phylophlan v3.0.2 based on the complete genome sequence, and the results prove that KRS010 aggregated into a single branch with *Bacillus altitudinis* GR-8^T^ (Fig. 2C). In addition, KRS010 has high average nucleotide identity (ANI) with *B. altitudinis* SORB11, GLB197, P-10, FD48, GR_8, SGAir0031, and DSM26896 at 98.62, 98.46, 98.45, 98.41, 98.40, 98.36, and 98.36%, respectively. The digital DNA-DNA hybridization (dDDH) values of all seven published *B. altitudinis* genomes against KRS010 were greater than 85% [28]. Taken together, the strain KRS010 was identified as *Bacillus altitudinis*.

### Physiological and biochemical characteristics of KRS010

The physiological and biochemical characteristics of KRS010 were summarized in Additional file 1: Table S2. Tricalcium phosphate and potassium solubilization; nitrogen fixation; active enzymes test such as oxidase, amylase, and catalase; gelatin liquefaction activity; citrate utilization; nitrate reduction; and glucose decomposition are all positive. Inorganic phosphorus solubilization, siderophore production, indole test, proteinase activity, phenylalanine deaminase activity, methyl red, and anaerobic determination are negative.

**Fig. 3:**
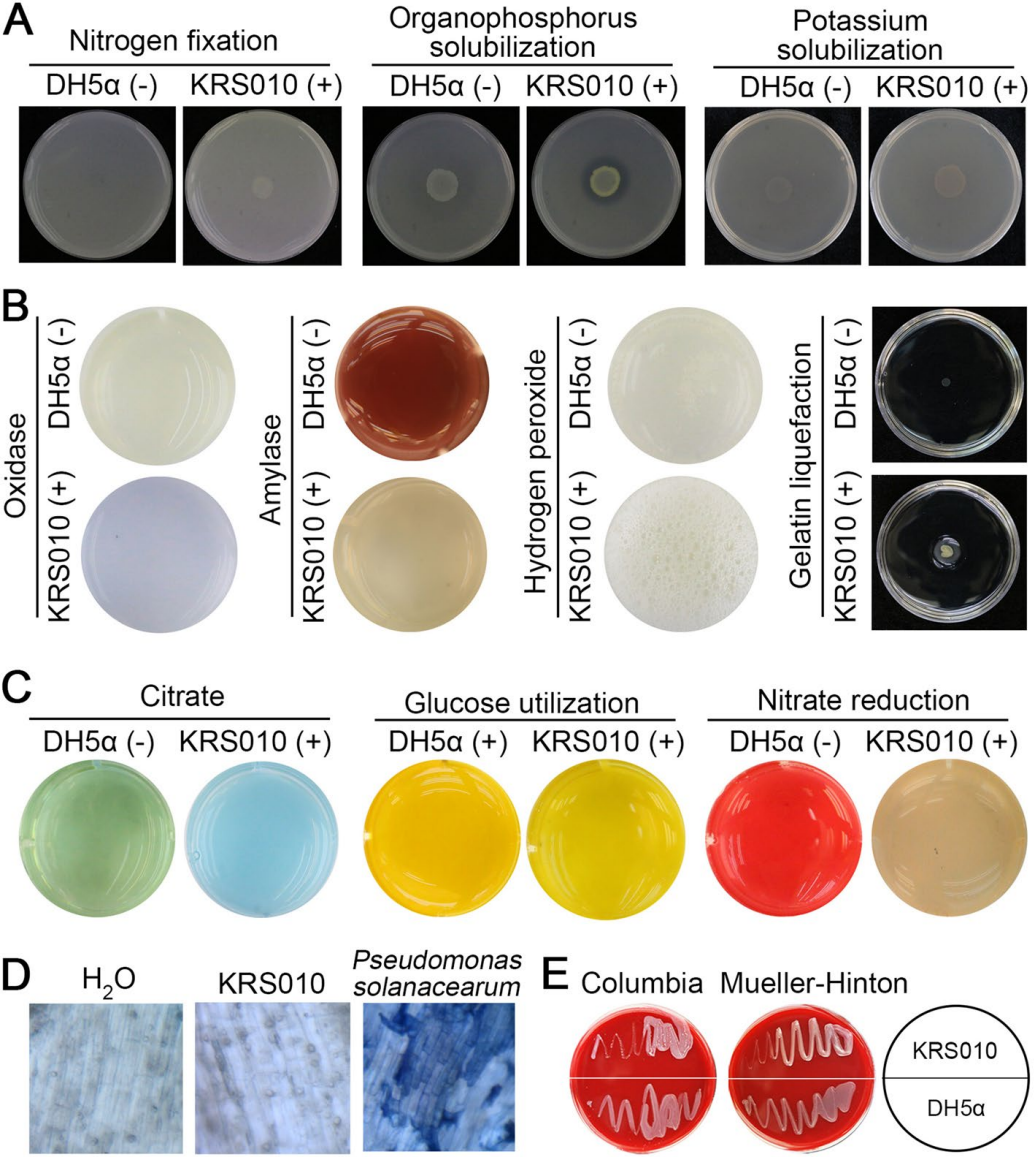
Analysis of physiological and biochemical characteristics in *B. altitudinis* KRS010. **A** Determination of relevant indicators for promoting growth. **B** Enzyme assay of strain KRS010. *E. coli* DH5a was severed as the negative control. **C** Determination of other physiological and biochemical indicators of strain KRS010. **D** Safety evaluation of strain KRS010 on cotton roots. **E** Hemolysis test using Mueller-Hinton (MH) plates and Columbia blood plates, and the *E. coli* DH5α was severed as control

Macronutrients nitrogen (N), phosphorus (P), and potassium (K) are the three most essential nutrients for plant growth and development, whereas only minor portions of them are available for the plant [29]. The isolate KRS010 had the strong ability of tricalcium phosphate and potassium solubilization and nitrogen fixation, which indicated this strain has a promising potential for plant growth promotion by providing an abundance of elements (Fig. 3A; Additional file 2: Fig. S3A). KRS010 also could produce a variety of enzymes such as oxidase, catalase, amylase, and gelatinase, which may destroy the phytopathogen’s cell wall to exert antifungal function and provide some available energy sources for itself (Fig. 3B; Additional file 2: Fig. S3B). Moreover, KRS010 is an aerobic bacterium that can use glucose and citrate as carbon sources (Fig. 3C; Additional file 2: Fig. S3C).

The safety of strains is an important precondition for the potential application of biocontrol microorganisms. The pathogenicity and hemolysis tests were performed to evaluate the safety of strain KRS010. The microscopic observation of cotton seedling root tissue treated by KRS010 fermentation broth showed that the root cells were not stained by the trypan blue dye (Fig. 3D). On the contrary, *Pseudomonas solanacearum*, as a pathogen of bacterial wilt, can cause the plant cell death, and these dead cells were stained blue using trypan blue dye (Fig. 3D). These results suggested KRS010 is not pathogenic to plants. For the sake of human and animal safety and health, the hemolytic test was conducted on Columbia and Mueller-Hinton blood plates in vitro. The hemolytic ring was not observed around the colony of KRS010, which suggested that the strain has no *α*-hemolysin and does not break down red blood (Fig. 3E). The above results provide the security evidence of strain KRS010 for further biocontrol application.

### KRS010 protects the plant against *Verticillium dahliae*

**Fig. 4:**
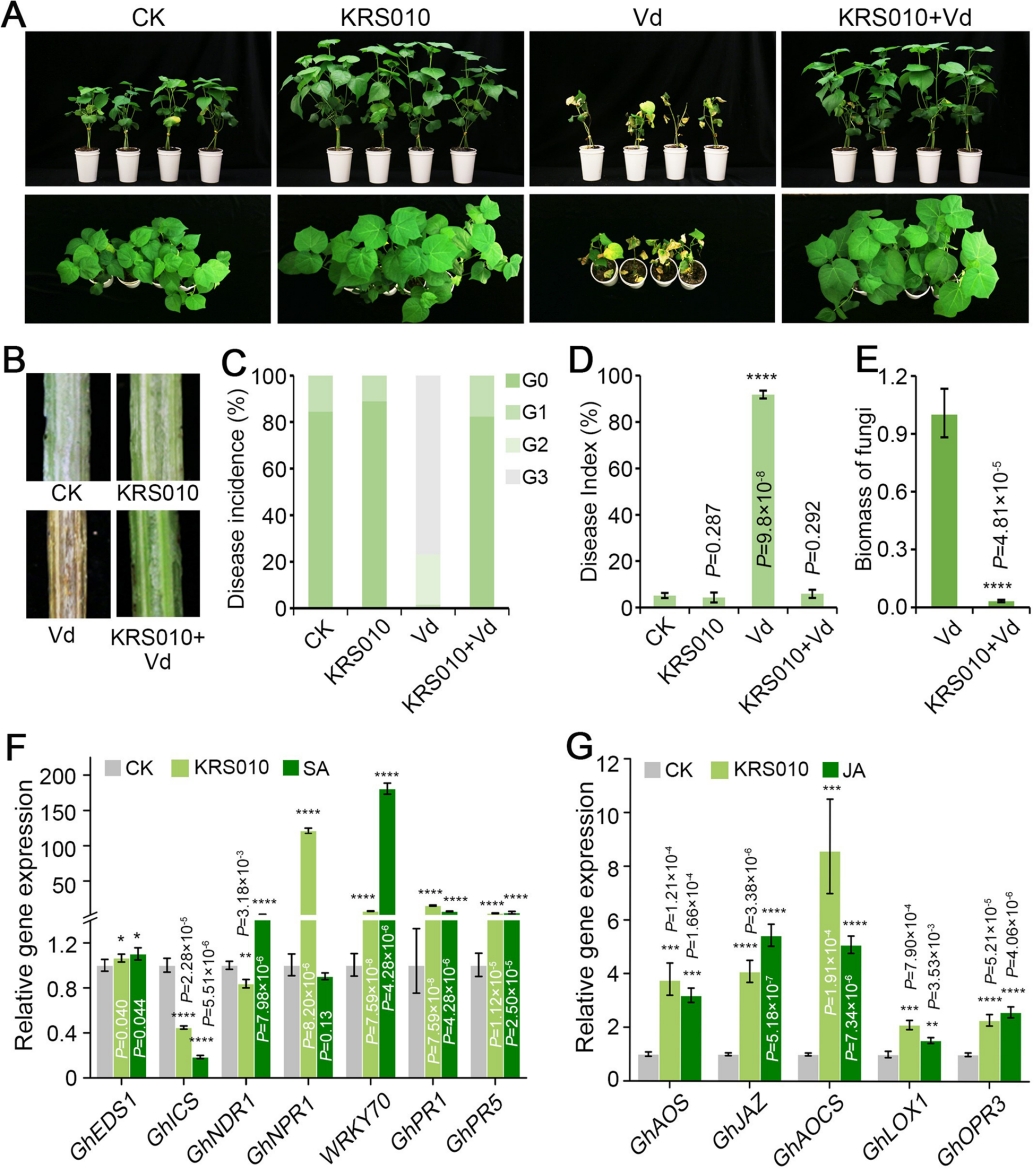
The strain KRS010 reduced the pathogenicity of *V. dahliae* in upland cotton. **A** Photos showing 3-week-old cotton plants grown in normal soil with CK (water), KRS010 (KRS010 fermentation broth with OD_600_ as 1.0), Vd (1 × 10^7^ conidia/mL suspension of *V. dahliae*) or KRS010+Vd (cell suspension of KRS010 with OD_600_ as 1.0 was irrigated, and 1 × 10^7^ conidia/mL suspension of *V. dahliae* was inoculated after 2 days). **B** Stem longitudinal sections of corresponding cotton seedlings. **C** Disease incidence of cotton seedlings. **D** Disease indexes of cotton seedlings. **E** Fungal biomass of *V. dahliae* on cotton was determined by quantitative PCR (qPCR). **F** The marked gene expression levels of the salicylic acid (SA) signaling pathway in cotton leaves. **G** The marked gene expression levels of the cotton jasmonic acid (JA) signaling pathway in cotton leaves. Error bars represent standard errors. **, ***, and **** represent significant differences at *P* < 0.01, *P* < 0.001, and* P* < 0.0001, respectively, according to the unpaired Student’s* t* test

The biocontrol effect of strain KRS010 against cotton Verticillium wilt was detected by a potted planting experiment. The four groups of cotton seedlings were prepared and were treated with the water treatment as the control (namely “CK”), the KRS010 fermentation broth (namely “KRS010”) only, the pathogenic fungus *V. dahliae* (namely “Vd”) only, and the KRS010 fermentation broth and *V. dahliae* (namely “KRS010+Vd”). Comparing with the “CK” group, the cotton seedlings grew slowly, the leaves presented yellow and wilt, and even fell off in the “Vd” group; the “KRS010” exhibited strong growth and was more resistant to Verticillium wilt than the control plants (Fig. 4A). It is worth noting that the cotton seedlings of “KRS010+Vd” group not only had no Verticillium wilt phenotype, but also grew better than the control group (Fig. 4A). As showed in Fig. 4B, only with the “Vd” group of the longitudinal section of vascular bundle in cotton appeared browning, but all of the “CK,” “KRS010,” and “KRS010+Vd” groups had no browning. The incidence and disease index of the cotton treated by conidia suspension of *V. dahliae* were 98.59% and 91.79, respectively, while those of applying the conidia suspension of *V. dahliae* after treatment with KRS010 fermentation broth were 18.66% and 5.88, respectively (Fig. 4C, D). The control efficiency of KRS010 against Verticillium wilt of cotton was 93.59%. The fungal biomass detection revealed that the isolate KRS010 fermentation broth resulted in reduced fungal growth compared to the “Vd” group (Fig. 4E). Taken together, the strain KRS010 could protect cotton seedlings from the infection of *V. dahliae*.

Biocontrol microorganisms could trigger an immune response in plants by producing secondary metabolites, which can enhance the ability of plants to resist biotic stress like pathogenic fungi. To investigate whether KRS010 could trigger the plant defense response, the related gene expression levels of salicylic acid (SA) and jasmonic acid (JA) signaling pathways were examined by RT-qPCR. Compared with water-treated control, these key genes of the SA signal pathway as *GhEDS1*, *GhNDR1*, *WRKY7*, *GhPR1*, and *GhPR5* were upregulated in cotton leaves after treating with 10 mmol/L SA. Similarly, the marked genes of SA signal pathway as *GhEDS1*, *GhNPR1*, *WRKY7*, *GhPR1*, and *GhPR5* were upregulated after treating with KRS010 fermentation broth (Fig. 4F). Simultaneously, compared with the water-treated control, both the treatment of KRS010 fermentation broth and 100 μmol/L JA can upregulate the expression of marked genes (*GhAOS*, *GhJAZ*, *GhAOCS*, *GhLOX1*, and *GhOPR3*) of JA signal pathway (Fig. 4G). In addition, compared with the control of water treated cotton, the marked genes of the SA signal pathway as *GhEDS1*, *GhICS*, *GhNPR1*, *WRKY7*, *GhPR1*, and *GhPR5* (Additional file 2: Fig. S4A) and the JA signal pathway as *GhAOS*, *GhAOCS*, *GhLOX1*, and *GhOPR3* (Additional file 2: Fig. S4B) were upregulated in cotton roots after irrigating with KRS010 fermentation broth for 2 days.

The indispensable role of SA in plant defense has been confirmed through studies conducted in SA-deficient tobacco and *Arabidopsis thaliana*. These transgenic plants express the bacterial *NahG* gene encoding a salicylate hydroxylase enzyme that converts SA to catechol; thus, these plants cannot accumulate significant amounts of SA. As a result, these plants exhibited enhanced susceptibility to viral, fungal, and bacterial pathogens [30, 31]. In our study, the expression levels of SA pathway-related genes (*NbPR1*, *NbPR2*, *NbPR5*, and *NbPAL*) were assessed using RT-qPCR after treating *NahG* transgenic tobacco leaves with the KRS010 fermentation broth or the 10 μmol/L SA solution, respectively. The results indicate that both KRS010 fermentation broth treatment and SA treatment can stimulate the upregulation of the key genes involved in the SA signal pathway (Additional file 2: Fig. S4C). Based on these results, it can be inferred that KRS010 may trigger plant immunity by inducing the upregulation of SA and JA signaling pathway-related genes, potentially contributing to its effective inhibition of Verticillium wilt occurrence.

### KRS010 inhibits conidia production, hyphal development, and melanin formation

**Fig. 5:**
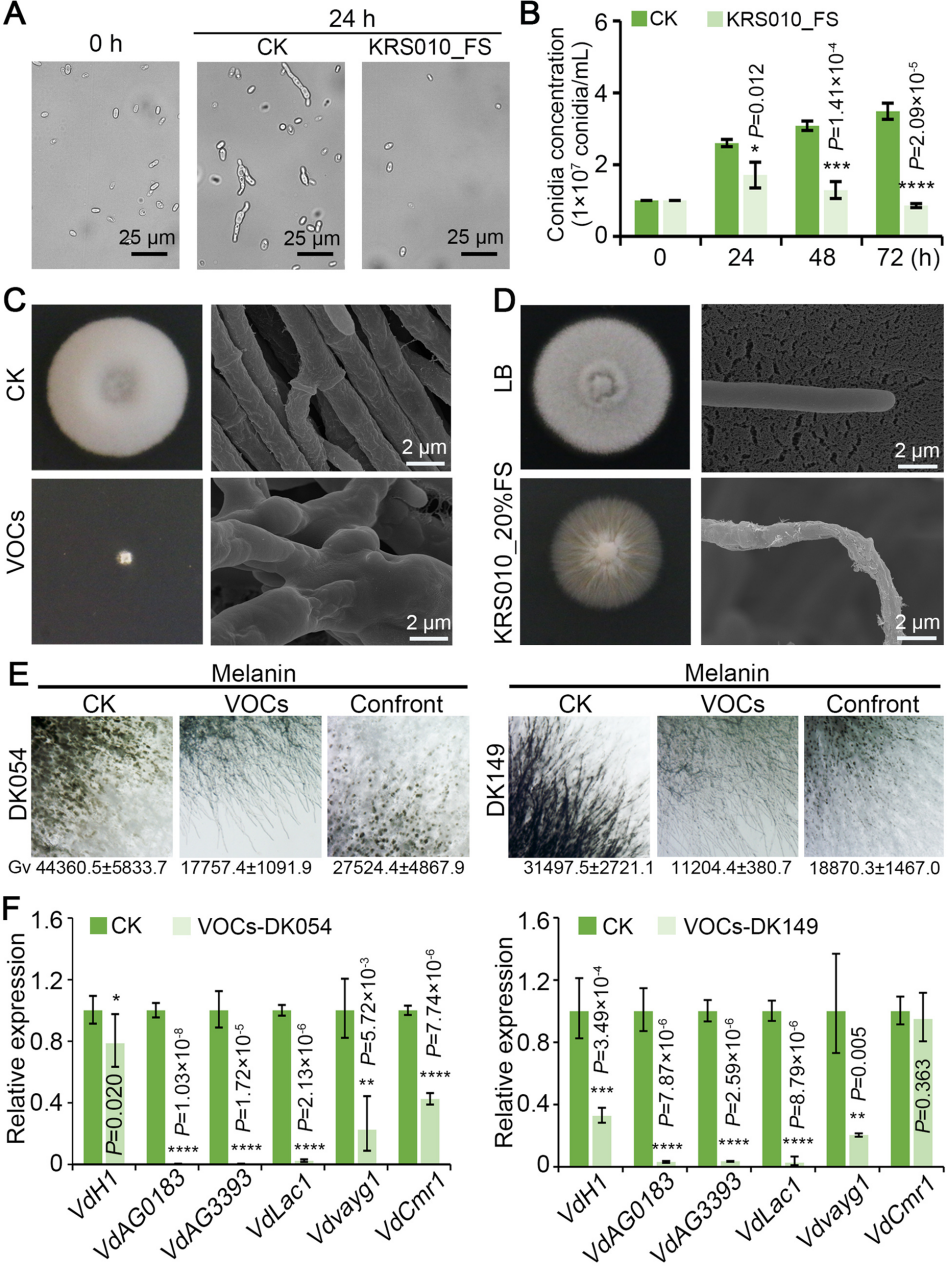
*B. altitudinis* KRS010 inhibits conidia production, hyphal development, and melanin production. **A** The mycelium morphology of *V. dahliae* in the presence of KRS010 fermentation broth for 24 h was observed by a differential interference contrast microscope (DIC). The mycelium incubated in LB broth was set as a control. **B** Conidia production statistics. **C**, **D** SEM was used to examine the hyphal morphology defects from the edges and superficial state of *V. dahliae* due to VOCs and the secondary metabolites of isolate KRS010, respectively. The bars represent the actual sizes. **E** The melanin production edges of DK054 and DK149 were observed by stereomicroscope. The numbers below represent the statistics of grayscale area values. **F** Relative expression of marker genes of *V. dahliae* melanin biosynthesis on the covering fumigation of KRS010 at 5 days. Error bars represent standard errors. **, ***, and **** represent significant differences at *P* < 0.01, *P* < 0.001, and *P* < 0.0001, respectively, according to the unpaired Student’s* t* test

Conidial germination is one of the factors to affect the development and prevalence of plant pathogens [32]. Hence, conidia production ability was determined to assess the inhibitory efficacy of KRS010 against *V. dahliae*. We evaluated the effect of different processing times of KRS010 culture filtrate on conidial germination of *V. dahliae*, and the initial concentration of the conidia suspension was 1 × 10^7^ conidia/mL. After 24 h, the conidia had no normal germination under the treatment of KRS010 culture filtrate by microscope observation (Fig. 5A). After 48 h and 72 h, all treatments showed a distinct difference in the germination rate compared with the control, and as the processing time increased, the conidial concentration gradually decreased (Fig. 5B).

The hyphal morphology of *V. dahliae* was observed by a scanning electron microscope (SEM) on PDA plates with 20% supernatant of extracellular metabolites of KRS010 or VOCs produced from KRS010 after 4 days. The results demonstrated that the hyphal morphology at the edges of the inhibitory zone showed enlargement, deformation, and aberrant dendritic branch after VOC treatment, while hyphae from the control group were intact and had normal radial growth (Fig. 5C; Additional file 2: Fig. S5B). The hyphal developmental morphology of *V. dahliae* was observed on PDA plates with 20% supernatant (extracellular metabolites of KRS010). Compared with the control, the hyphal was disorderly grown, dissociated, hollows, and crystals formed on the surface. The hyphae in the control group were normal, with smooth, plump, and well-rounded surfaces (Fig. 5D; Additional file 2: Fig. S5C). In general, mycelial morphology observed with SEM has been severely altered.

### B. altitudinis KRS010 disturbs the melanin formation of *V. dahliae*

Melanin is essential in *V. dahliae* for the development of fully functional microsclerotia [33]. The production of melanin may be critical for long-term survival, as melanin allows *V. dahliae* and other fungi to protect against damage from UV irradiation, extreme temperatures, and enzymatic degradation by soil microorganisms. In wild-type *V. dahliae*, the presence of melanin implies the presence of microsclerotia, and its absence implies the absence of microsclerotia [34]. We further studied the melanin production of strain KRS010 in controlling fungi. The melanin production of *V. dahliae* was observed by stereomicroscope on PDA plates with confrontation culture and the covering fumigation of KRS010. The results demonstrated that inhibition of mycelial growth and reduction of melanin production in the treated group (Additional file 2: Fig. S5A). The relative transcript levels of genes involved in *V. dahliae* melanin synthesis were detected under confrontation culture and covering fumigation between KRS010 and *V. dahliae* by RT-qPCR. Genes related to melanin synthesis include *VdVayg1*, *VdAG_00183*, *VdAG_03393*, *VdCmr1* [35], *VdLac1*, and *VdH1*, which are necessary for melanin and microsclerotium production. Compared with the control, the marker genes of melanin biosynthesis (*VdAG_00183*, *VdAG_03393*, *VdVayg1*, and *VdLac1*) were downregulated obviously after 4 days of treatment through covering fumigation of KRS010 (Fig. 5F). Taken together, the strain KRS010 had the ability to prevent melanin synthesis that was caused by both supernatant compounds and VOCs.

### B. altitudinis KRS010 promotes plant growth

**Fig. 6:**
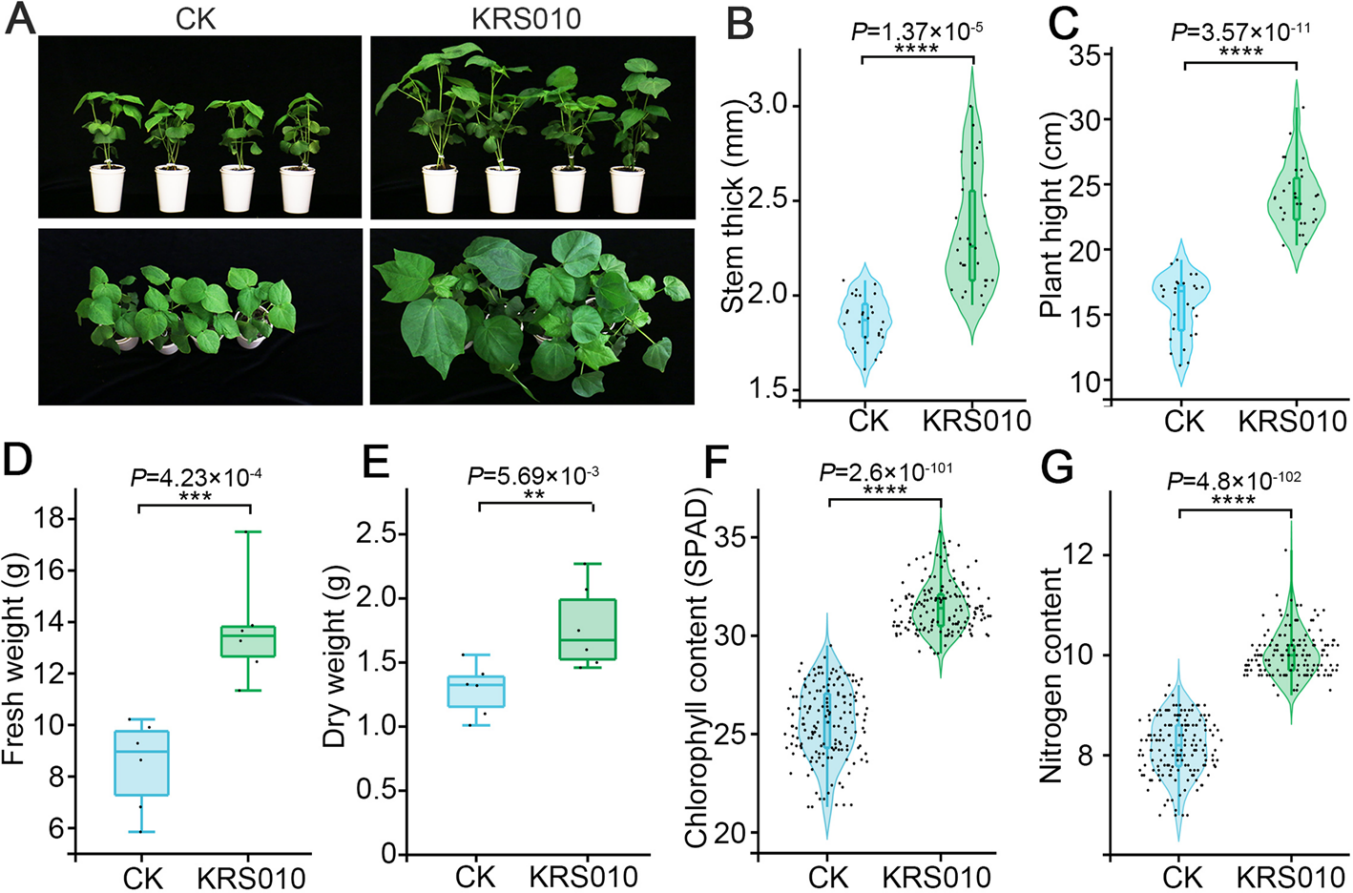
The strain KRS010 promoted plant growth. **A** Photos showing 3-week-old cotton plants grown with CK (water) and KRS010 (KRS010 fermentation broth with OD_600_ as 1.0). Each experiment included four repetitions and was repeated three times. **B** Shoot stem thick of plants. **C** Shoot height of plants. **D** Shoot fresh weight of plants. **E** Shoot dry weight of plants. **F** Leaf chlorophyll content of plants. **G** Leaf nitrogen content of plants. One-way analysis of variance (ANOVA) was used to analyze the statistical significance of multiple groups

Plant growth-promoting bacteria (PGPB) are a promising alternative to conventional fertilization. One of the most interesting PGPB strains is *B. altitudinis*. It is a bacterial species that inhabits a wide range of environments or plants [36]. In the current study, the effect of *B. altitudinis* KRS010 (OD_600_ = 1.0) on the growth of cotton was explored (Fig. 6A). Compared with the control (CK), KRS010 markedly increased the stem thick (Fig. 6B) and plant height (Fig. 6C) of cotton in the treatment. KRS010 also increased fresh weight (Fig. 6D) and dry weight (Fig. 6E) of cotton. The results showed that the chlorophyll (Fig. 6F) and N content (Fig. 6G) increased after treating with KRS010 fermentation broth. In summary, KRS010 fermentation broth can promote the growth of cotton plants.

## Discussion

Verticillium wilt occurs worldwide in cotton and is difficult to manage. Biocontrol has been proposed as an environmentally friendly, sustainable, and effective approach to control plant diseases, but study on the use of biocontrol for Verticillium wilt is limited. Endophytes are plant-associated microorganisms that live in plant internal tissues in a similar niche as phytopathogens and may compete as biocontrol agents with pathogens [37]. It has been reported that *B. altitudinis* GLB197 exhibited a strong antagonistic activity against various plant pathogens especially for the grape downy mildew, indicating that *B. altitudinis* are prospective bacteria for controlling fungal diseases [38]. In the current study, strain KRS010 was isolated from the seed of Verticillium wilt-resistant *G. hirsutum* cultivar “Zhongzhimian No.2.” This bacterial strain KRS010 was identified as *B. altitudinis* based on its morphology and molecular characteristics.

Before using the isolated strains for biocontrol or biofertilization purposes, it is essential to ensure their safety and harmlessness. In case of *B. altitudinis* KRS010, it has been determined to have no phytotoxic effects on cotton roots (Fig. 3D) and cause hemolysis of red blood cells in the conducted hemolysis test (Fig. 3E). Therefore, it appears that *B. altitudinis* KRS010 is harmless for both plants and humans. Nonetheless, it is crucial to conduct further investigation to determine its pathogenicity in humans, including acute toxicity test using appropriate methodologies and hereditary toxicity tests.

Root exudates of an appropriate host induce germination of Verticillium microsclerotia and hyphal growth towards host plant roots [39]. Destroying the cell wall of plant pathogens is one of the effective ways for biocontrol bacteria to exert antifungal activity [40]. In this study, strain KRS010 was characterized by its potential to produce various enzymes involved in pathogen hyphae degradation, including oxidase, amylase catalase, gelatinase, and hydrogen peroxide (Fig. 3B). Through spore germination experiments, the conidia production of *V. dahliae* decreased after treatment with KRS010 cell-free supernatant (Fig. 5B). This suggests that KRS010 metabolite may damage or destroy fungal conidia or germinating hyphae of *V. dahliae*. Moreover, it is demonstrated that the hyphae of *V. dahliae* were deformed and destroyed in the covering fumigation culture treated with KRS010 (Fig. 5C, D). In summary, KRS010 has the ability to destroy the cell wall of pathogens, possibly through synthesizing relevant enzymes.

Through a variety of mechanisms, including the suppression of pathogens by the production of antibiotics, occupation for niches within the rhizosphere, and alteration of the rhizosphere microbial community structure, beneficial plant-microbiome interactions improve plant fitness through growth promotion [41]. Importantly, the production of biofilm by root bacteria is a critical stage for the colonization of microorganisms in the plant root [42]. Biofilms are communities of surface-attached bacteria that are enveloped in extracellular matrix [43]. Exopolysaccharides (EPS) and the amyloid-forming protein TasA are the main components of biofilm produced by *Bacillus* species [44]. *Bacillus velezensis* has been reported to form biofilm for recruiting beneficial microorganisms from the cucumber rhizosphere and jointly promoted plant growth and helped plants alleviate salt stress, and synergistic biofilm formation was accompanied by enhanced plant growth-promoting and salt stress-relieving ability [45]. The target strain KRS010 forms biofilm in static LB liquid medium (Fig. 2C). Moreover, the plant growth promotion traits were evidenced in the greenhouse when the strain KRS010 was inoculated into plants (Fig. 6A). The impacts on cotton plant seedling length, root length, and fresh weight were all clearly enhanced. The growth-promoting ability of KRS010 may be related to biofilm formation. Moreover, KRS010 could solubilize phosphates and potassium and have a nitrogen fixation function (Fig. 3A), which offered competitive advantages for biological control application and suggested that *B. altitudinis* KRS010 might have a growth-promoting function.

Melanin is produced by a broad variety of pathogenic microorganisms, and its production may be stimulated in stress responses, including bacteria, fungi, and helminths [46]. Fungal melanin seems to be essential for the survival of propagative cells during protracted growth-unfavorable periods. Melanin is found as dense granular screens in the cell wall and the matrix surrounding the wall [47]. Melanin deposition is tightly coupled with microsclerotia development of *V. dahliae* and provides protection against adverse environments. In previous studies, *VdCmr1* within the melanin biosynthetic gene cluster in *V. dahliae* strain VdLs.17 revealed that it was required for full virulence on lettuce and tobacco [48] and was required for survival in response to UV irradiation and high-temperature stress [49]. Many genes upstream of the melanin biosynthesis pathway also influence its biosynthesis in *V. dahliae*. Enzyme Vayg1 in the DHN pathway harbors an esterase/lipase (Aes) domain, and VdVayg1 catalyzes the transformation of melanin precursor heptaketide naphthopyrone (YWA1) to 1,3,6,8-THN. It has been reported that deletion of the *Vayg1* gene hindered not only the melanin production but also the microsclerotial formation [50]. Due to its requirement for the production of wild-type microsclerotia and greater desiccation tolerance in conidia, *VdH1* is thought to be crucial to the persistence of the pathogen in soils and the spread of the disease [51] *VdAG_00183*, *VdAG_03393*, and *VdLac1* participate in *V. dahliae* melanin formation. Marker genes (*VdCmr1*, *Vayg1*, *VDH1*) involved in melanin synthesis were suppressed under the condition of dual confrontation culture between KRS010 and fungi (Fig. 6C). Thus, the inhibition of melanin formation by KRS010 could also be one of the possible mechanisms for direct fungal control.

In this study, we found that strain KRS010 has the control effect of suspension on the whole cotton plant against Verticillium wilt (Fig. 4A, B), which was similar with previous study [52]. *Bacillus* strains T6 has been proved to reduce disease severity of cotton Verticillium wilt by 92.55% under greenhouse conditions [53]. Although the inhibitory effect of KRS010 fermentation broth on Verticillium wilt was significant in greenhouse experiments, its effectiveness has not been validated in field experiments. Further experiments can be conducted for in-depth studies to confirm its potential practical application in agriculture. It has been also demonstrated that biocontrol microorganisms can increase the ability of plants to withstand biotic stress by inducing systemic resistance (ISR) and plant immunity through the release of secondary metabolites. The defense hormones salicylic acid (SA) and jasmonic acid (JA) serve as defensive signals by regulating the expression of genes involved in defense and promoting plant disease resistance. SA is a beta-hydroxy phenolic acid that has been widely known as a defense-related phytohormone [54]. SA is one of the essential hormones connected to defense that activates the immune system’s defenses against pathogens. The primary function of SA is the development of systemic acquired resistance (SAR), a durable and all-encompassing type of disease resistance [55]. JA regulates plant defense against necrotrophic pathogens. Fungal pathogens like *Alternaria brassicicola*, *Botrytis cinerea*, *Sclerotinia sclerotiorum*, *Plectosphaerella cucumerina*, and *Fusarium oxysporum* are examples of necrotrophic pathogens that are impacted by JA-induced plant defenses [44]. It has been found in our study that treatment with KRS010 fermentation broth increased the expression of SAR marker genes such as *GhEDS1*, *GhICS*, *GhNPR1*, *GhNDR1*, *WRKY7*, *GhPR1*, and *GhPR5* in cotton leaves, which suggested that KRS010 stimulates the SAR in the host plant (Fig. 4F). At the same time, the transcriptional levels of JA-related marker genes were increased under the treatment of KRS010 fermentation broth (Fig. 4G). Likewise, recent research has shown that *Bacillus amyloliquefaciens* CRN9 inhibits pathogen colonization and helps them to resist early cotton diseases by inducing ISR that is related to both the SA- and JA/ET-dependent signaling pathways [56]. Combined with our results, the SA- and JA-dependent signaling pathways may participate in *B. altitudinis* KRS010-induced ISR in cotton, thereby reducing the occurrence of Verticillium wilt.

The antimicrobial compounds produced by *Bacillus* species either directly hamper the growth of the pathogen or ISR in plants [57]. *Bacillus* species have exhibited the activity of pathogen inhibition by secretion of secondary metabolites such as pyrrolnitrin, phenazines, siderophores, nonribosomal peptides and polyketides, phenazine-1-carboxylic acid, and volatile organic compounds (VOCs). For example, the lipopeptide of *B. altitudinis* Q7 inhibits the growth *Alternaria alternata* [58]. Gotor-Vila et al. (2017) found the use of *B. amyloliquefaciens* VOCs in the control of *Monilinia laxa*, *Monilinia fructicola*, and *B. cinerea* in post-harvest sweet cherry fruits, thereby reducing the incidence of disease and sporulation of pathogens [59]. The antifungal active substances of strain KRS010 might be disclosed by the whole genome of strain KRS010 combined with liquid chromatography-mass spectrometry analysis. KRS010’s complete genome analysis revealed that it harbors genes encoding numerous enzymes and has the capacity to break down the cell wall of pathogens [28]. In this study, KRS010 has been characterized as an excellent antifungal ability in both confrontation culture and covering fumigation assay (Fig. 1B, C). It is thus evident that the antifungal VOCs of KRS010 have potential application prospects for managing fungal disease in cotton. The successful commercial application of VOCs hinges on a comprehensive understanding of their antifungal mechanisms. Our preliminary results demonstrated that some potential bacteriostatic VOCs released from KRS010 were identified by gas chromatography-mass spectrometry (GC-MC), such as 1-butanol, 2-heptanone, 1-butanol, 3-methyl, 2-heptanone, 6-methyl, propanoic acid, 2-methyl, hexanoic acid, and methyl hexadecanoate. The detailed investigation and corresponding discussion will be presented in our next manuscript. Further, in-depth investigations are imperative to fully elucidate the antifungal mechanisms of KRS010 VOCs and explore their potential for preventing and controlling post-harvest diseases in fruits and vegetables, with the aim of developing antifungal compounds suitable for commercial applications.

## Conclusions

In conclusion, the strain KRS010 was identified as *B. altitudinis* by morphological identification, phylogenetic analysis, and physio-biochemical characteristics. The isolate has displayed broad-spectrum antagonistic activities in vitro. Meantime, KRS010 exhibited robust preventive effects against Verticillium wilt during potted plant control assay and triggered a plant immune response. The possible mode of these activities in this strain includes fungal cell wall degradation and inhibition of melanin biosynthesis. In summary, *Bacillus altitudinis* KRS010 is a beneficial microbe which provides a promising approach for managing cotton Verticillium wilt in the future.

## Methods

### Culture of microbes and plant material

The antagonistic bacteria KRS010 was isolated from the seed of Verticillium wilt-resistant *G. hirsutum* cultivar “Zhongzhimian No. 2” and incubated on Luria-Bertani (LB, tryptone 10 g, NaCl 10 g, yeast extract 5 g, double-distilled H_2_O 1000 mL) plates at 28 °C. The pathogenic fungi were cultured on potato dextrose agar (PDA, potato 200 g, glucose 20 g, agar 15 g, ddH_2_O 1000 mL) plates at 25 °C. *G. hirsutum* cultivar Junmian No. 1 cotton seedlings and *NahG* transgenic tobacco plants were cultured in 25 °C greenhouse with a 16-h light and 8-h darkness photoperiod. The *NahG* transgenic tobacco plants expressing a SA hydroxylase gene from *Pseudomonas putida* are able to metabolize SA to catechol, leading to a dramatic decrease in the plant SA content. Throughout the growing period, these cotton plants were cultivated in a fixed position in the greenhouse.

### Identification of antagonistic bacterium KRS010

#### Morphological identification

The strain KRS010 was streaked on an LB plate and cultured at 28 °C for 12 h, in which the morphology, color, wet or dry, smooth or rough of a single colony was observed. Gram staining was conducted by a series of steps including initial dyeing, mordant dyeing, decolorization, and redyeing by Gram Staining kit (Coolaber, SL7040) [60]. *Escherichia coli* DH5α was used as negative control. In order to observe the biofilm formation, a single colony of the KRS010 was inoculated in 50 mL of LB broth with 200 rpm at 28 °C overnight, followed by stationary incubation at 28 °C for 7 days for the formation of biofilm.

#### Molecular identification

A phylogenetic analysis of KRS010 and other *Bacillus* species strains was conducted with Phylophlan v3.0.2 based on the complete genome sequence. Phylogenetic tree was established by TVBOT (https://www.chiplot.online/circleTree.html).

#### Physiological and biochemical characteristics analysis

Physiological and biochemical characteristics were assessed according to the methods described in Bergey’s Manual of Systematic Bacteriology and the Common Bacterial Identification Manual [61, 62]. The experiment includes the characterization of capability for inorganic phosphorus utilization (Bacterial Inorganic Medium, Hopebio, HB8670), organic phosphorus utilization (Bacterial organophosphorus Medium, Hopebio, HB8673), potassium utilization (Silicate bacteria medium, Coolaber, MM6021), nitrogen fixation (Ashby’s Medium, COOLABER, MM5041), siderophore production (modified CAS agar medium kit, Coolaber, PM0821), IAA synthesis, oxidase activity, amylase activity, hydrogen sulfide production (Triple sugar iron agar, COOLABER, MM3011), gelatin liquefaction, phenylalanine deaminase test (Phenylalanine medium, Solarbio, LA1720), protease activity, methyl red test, gluconate production, nitrate reduction, citrate production, and anaerobic determination. All the above tests were performed twice, and each experiment was performed in triplicate.

#### Safety evaluation

The roots of cotton seedlings were soaked in KRS010 cell suspension (OD_600_ = 1.0) for 1 h, followed by staining with trypan blue for 3 h, and the roots were observed with a microscope. Roots soaking with LB broth were severed as control. Mueller Hinton (MH) plates and Columbia blood plates were used to detect the hemolysis of KRS010. The *E. coli* DH5α has no hemolysis [63] that was used as a negative control. All of these assays were repeated twice, and each treatment was performed in triplicate.

### Identification for antifungal activity of KRS010 in vitro

The activity of KRS010 against the colony growth of seven pathogenic fungi was evaluated through confrontation tests and the treatments of volatile organic compounds (VOCs). Firstly, seven pathogenic fungi were firstly cultured on the center of the PDA medium for 4 days, and the hyphal bulks with a diameter of 6 mm were inoculated to the center of the PDA plates.

For confrontation assays, the 10 μL KRS010 (OD_600_ = 1.0) cell suspension was dropped at 20 mm from the top, bottom, left, and right of the fungal bulks, respectively. The 10-μL LB broth at the same position of the hyphal bulks was severed as controls. All plates were incubated at 25 °C for 3~7 days, and the semidiameter of the pathogen mycelium disc grown on the treated plates and the control plates were measured by cross-measurement method. The semidiameter of the colony was recorded, of which the colony semi-diameters of the control and treatment group were marked as “A” and “B,” respectively. The inhibition rate was calculated by the following formula: inhibition rate (%) = [(A − B)/(A − 6)] × 100.

For the assays of VOCs released from KRS010 on mycelial growth, LB plates covering 100 μL KRS010 cell suspension were prepared, and these treated LB plates were sealed with the prepared plates with fungal bulks by parafilm. The LB plates without KRS010 cell suspension were used as control. All plates were incubated at 25 °C for 3~7 days, and the diameter of the colony was measured, of which the colony diameters of control and treatment were marked as “a” and “b,” respectively. The inhibition rate was calculated using the formula: inhibition rate (%) = [(a − b)/(a − 6)] × 100.

The cell-free culture filtrate was obtained as described in a previous study with some modifications [64]. KRS010 fermentation broth was centrifuged at 8000 rpm for 20 min, and the supernatant was collected and filtrated through a 0.22-µm microporous membrane and stored at 4 °C. The cell-free supernatant was mixed into PDA plates at the proportion of 5%, 10%, and 20% (v/v), respectively, and inoculated with fungal bulks of *V. dahliae*. The hyphal growth and development were observed after 5 days.

All the above experiments were performed twice, and each treatment/group in every experiment was conducted at least three plates.

### Biocontrol effect of KRS010 against cotton Verticillium wilt

Three-week-old cotton seedlings were used to evaluate the biocontrol activity of isolate KRS010 against Verticillium wilt caused by *V. dahliae*. The KRS010 cell suspension with OD_600_ = 1.0 and conidial suspension of *V. dahliae* as 1 × 10^7^ conidia/mL were prepared. This assay included four groups: cotton seedlings were treated with KRS010 cells suspension individually (marked with “KRS010”), conidial suspension of *V. dahliae* individually (marked with “Vd”), KRS010 cells suspension with conidial suspension of *V. dahliae* (marked with “KRS010+Vd”), and water only treatment (marked with “CK”). The amount of inoculation was 20 mL, and 20 mL of corresponding solvent was used for other treatments each time. All of the treatments were incubated at the same conditions as the previous cotton seedlings culture. Disease symptoms were observed and quantified at 2 days post-treatment following the method in our previous study [65]. Symptoms of the seedlings at 30 days were surveyed and classified into 5 levels: level 0, healthy seedlings; level 1, ≤ 25% of leaves showing chlorosis or wilt; level 2, ≤ 50% of leaves showing chlorosis or wilt; level 3, ≤ 75% of leaves showing chlorosis or wilt; and level 4, more than 75% of leaves showing chlorosis or wilt [66]. At least 16 cotton seedlings were used for each group in 4 pots, and the experiment was conducted 3 times. The disease index (DI) and the control efficacy were calculated with the following formulas [67, 68]:

$$\mathrm{DI}=\;\left[\sum\left(\mathrm{the}\;\mathrm{seedling}\;\mathrm{of}\;\mathrm{every}\;\mathrm{grade}\;\times\;\mathrm{relative}\;\mathrm{grade}\right)\;/\left(\mathrm{total}\;\mathrm{seedlings}\;\times\;\mathrm{the}\;\mathrm{most}\;\mathrm{serious}\;\mathrm{grade}\right)\;\right]\;\times\;100\;$$

$$\mathrm{Control}\;\mathrm{efficacy}\;(\%)\;=\left[\left(\mathrm{Disease}\;\mathrm{index}\;\mathrm{of}\;\mathrm{control}-\;\mathrm{Disease}\;\mathrm{index}\;\mathrm{of}\;\mathrm{treated}\;\mathrm{group})/\mathrm{Disease}\;\mathrm{index}\;\mathrm{of}\;\mathrm{control}\right)\right]\;\times\;100$$

Subsequently, the stems of the seedlings were slit longitudinally to check the damage caused by *V. dahliae*, and the roots of the seedlings were collected for *V. dahliae* biomass analysis.

The genomic DNA (gDNA) of cotton was extracted according to a DNAsecure plant kit (Tiangen, Beijing, China) for the detection of relative fungal biomass. SYBR green-based qPCR was used to detect the biomass of *V. dahliae* with an initial 95 °C denaturation step for 3 min, followed by denaturation for 15 s at 95 °C, annealing for 20 s at 60 °C, and extension for 20 s at 72 °C for 34 cycles. The *V. dahliae EF1α* gene was used to quantify fungal colonization, and the *Ubiquitin* gene served as an endogenous control. These primer pairs are listed in Additional file 1: Table S1. Moreover, the plant height, stem thickness, dry weight, fresh weight, chlorophyll content, and nitrogen content of cotton in four treatments were also compared to estimate the growth-promoting effect of KRS010. Every experiment was performed with the same batch of cotton seedlings. At least 20 cotton seedlings were treated for each treatment, and the experiment was conducted three times.

### RT-qPCR for the relative gene expression levels in cotton or *V. dahliae*

The expression levels of defense-related genes in cotton roots and leaves were detected by reverse transcription quantitative PCR (RT-qPCR). The KRS010 fermentation broth (OD_600_ = 1.0), the solution of 10 mmol/L SA, and the solution of 100 μmol/L JA was sprayed on the 3-week-old cotton leaves for 12 h before collecting leaves [69]. The KRS010 fermentation broth (OD_600_ = 1.0) was inoculated on the 3-week-old cotton roots for 48 h before collecting the roots. The water treatment group was severed as a control. For the detection of defense-related gene expression levels in SA-deficient tobacco, the water, the KRS010 fermentation broth (OD_600_ = 1.0), and the solution of 10 μmol/L SA were sprayed on the 3-week-old tobacco leaves for 6 h before collecting leaves [70].

Total RNA extraction and first-strand cDNA synthesis were performed using the EASYspin Plus RNA speed extract kit (Aidlab, Beijing, China) and a cDNA synthesis supermix kit (TransGen, Beijing, China) according to the manufacturer’s instructions (both kits included a gDNA-removal procedure). For cotton, gene expression levels of the salicylic acid (SA) pathway-related genes (*GhEDS1*, *GhICS, GhNPR1*, *GhNDR1*, *WRKY7*, *GhPR1*, and *GhPR5*) and jasmonic acid (JA) pathway-related genes (*GhAOS*, *GhJAZ*, *GhAOCS*, *GhLOX1*, and *GhOPR3*) were normalized to the *GhUBQ7* gene by RT-qPCR. For tobacco, gene expression levels of the salicylic acid (SA) pathway-related genes (*NbPR1*, *NbPR2*, *NbPR5*, and *NbPAL*) were determined by RT-qPCR after normalizing to the *NbEF-1α* gene.

For the detection of hypha melanin biosynthesis-related gene expression levels in *V. dahliae*, the 100-mL cell suspension of KRS010 (OD_600_ = 1.0) was coated on an LB plate, and 100 mL conidial suspension of *V. dahliae* was inoculated on an organic filter membrane on top of a PDA plate. Then, the two treated plates were sealed together with parafilm and incubated at 25 °C for 6 days. The two sealed base plates, involving a LB plate with 100 mL LB broth and a PDA plate with *V. dahliae*, served as control. All treatments and controls were performed with five replicates. The fungal hypha was collected from the organic filter followed by total RNA extraction and synthesis of first-strand cDNA. The melanin biosynthesis and autophagy processes genes (*VdVayg1*, *VdAG_00183*, *VdAG_03393*, *VdCmr1*, *VdLac1*, and *VdH1*) were detected and normalized to the *V. dahliae EF1α* gene by RT-qPCR.

Reverse transcription-quantitative PCR (RT-qPCR) was carried out using TransStart Top Green qPCR SuperMix (+DyeII) (TransGen, Beijing, China) following the manufacturer’s instructions. Relative transcript levels of different genes among various samples were evaluated using the 2^−∆∆CT^ method as described previously [71]. The RT-qPCR experiment was repeated twice, and each contained three technical replicates. The RT-qPCR primer pairs are listed in Additional file 1: Table S1.

### Microscopic observations

After 4 days of treatment of VOCs or confrontation culture of KRS010, the hyphae at the edge area of *V. dahliae* were observed via a stereomicroscope (Nikon SMZ18). The grayscale area is used to evaluate the production of melanin using ImageJ calculation.

Strain KRS010 was incubated in LB at 28 °C for 96 h with shaking (180 rpm) and the cultural liquid was centrifuged to separate a supernatant and precipitate at 4 °C and 8000 rpm for 15 min. The supernatant was filtrated through a bacterial filter (0.22 μm), and the aseptic filtrate was obtained. After the conidial suspension of *V. dahliae* was incubated with cell-free culture filtrate of KRS010, conidial of *V. dahliae* was observed via a differential interference contrast microscope (DIC) at 12 h, 24 h, and 48 h. After 4 days of treatment with VOCs and a 20% cell-free supernatant of KRS010, the hyphae at the edge of the bacteriostatic area were picked with an inoculation blade and placed in a sterile centrifuge tube that contained 2.5% glutaraldehyde fixative for 48 h. These samples were observed with a scanning electron microscope (SEM). Before observations, the pretreated samples were dehydrated using graded ethanol at 30%, 50%, 70%, 80%, 90%, 95%, and 100%, respectively. The samples were dried using a CO_2_ critical point dryer.

### Statistical analysis

The standard errors in all involved figures were calculated for each treatment or group with at least three replicates. Unpaired Student’s *t* test was performed to determine statistical significance. One-way analysis of variance (ANOVA) and least significant difference (LSD) were used to analyze the statistical significance of multiple groups.

### Supplementary Information

Additional file 1: Table S1. Primers used in this study. Table S2. Plant growth-promoting (PGP) characteristics and physiological and biochemical characteristics assessment of the strain KRS010.

Additional file 2: Fig. S1. The broad-spectrum inhibition activity of strain KRS010 against phytopathogenic fungi. Fig.e S2. Morphological identification of KRS010. Fig. S3. Analysis of physiological and biochemical characteristics in B. altitudinis KRS010. Fig. S4. Relative expression of marker genes in plant treated with KRS010 detected by RT-qPCR. Fig. S5. KRS010 inhibits hyphal development and melanin production of V. dahliae.

Additional file 3. Individual data points.

## Data Availability

All study data are included in the article and/or supplementary information.
