# Supplementary material for: Insights into the biocontrol and plant growth promotion functions of Bacillus altitudinis strain KRS010 against Verticillium dahliae
Source: BMC Biol. 2024 May 20;22:116. doi: 10.1186/s12915-024-01913-1 (PMC11103837; doi:10.1186/s12915-024-01913-1)
Supplement: Supplementary file 2 — Additional file 2: Fig. S1. The broad-spectrum inhibition activity of strain KRS010 against phytopathogenic fungi. Fig.e S2. Morphological identification of KRS010. Fig. S3. Analysis of physiological and biochemical characteristics in B. altitudinis KRS010. Fig. S4. Relative expression of marker genes in plant treated with KRS010 detected by RT-qPCR. Fig. S5. KRS010 inhibits hyphal development and melanin production of V. dahliae. [file 12915_2024_1913_MOESM2_ESM.docx]

# Supplementary Figures

## Figure S1


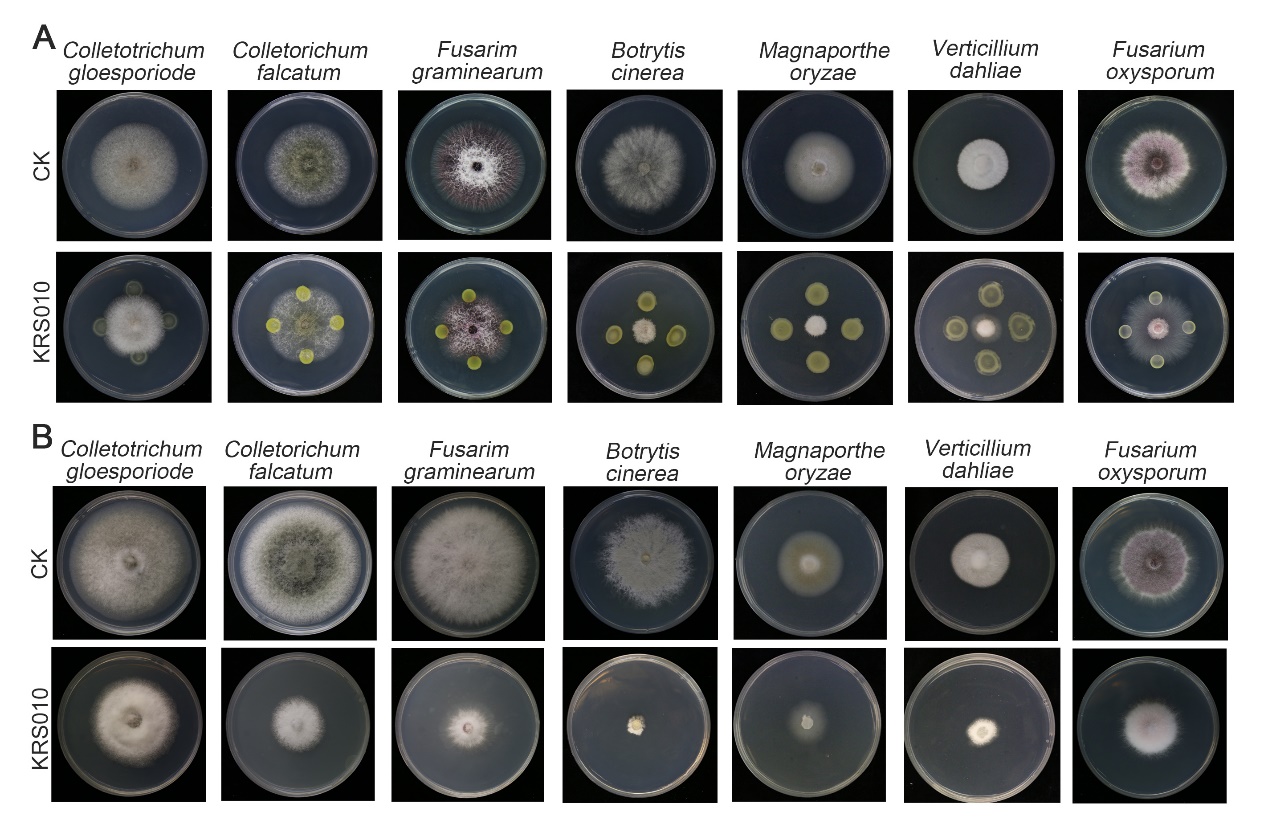


**Figure S1. The broad-spectrum inhibition activity of strain KRS010 against phytopathogenic fungi.** **(A)** Antifungal activity of strain KRS010 against seven phytopathogenic fungi by confrontation culture assay. **(B)** Antifungal activity of VOCs produced by KRS010 by the covering fumigation. The pathogenicity of each respective *VdPKS* was analyzed with three replicates of 20 cotton plants. Each treatment had three biological repeats.

## Figure S2


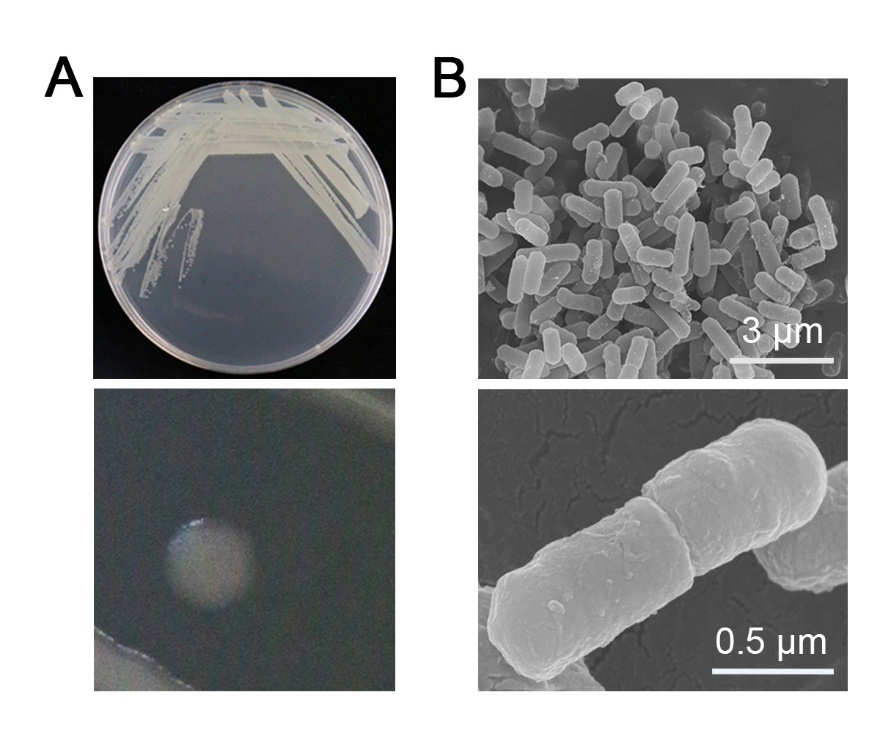


**Figure S2. Morphological identification of KRS010. (A)** The growth phenotype of strain KRS010 on LB medium. **(B)** Scanning electron microscopy (SEM) observation of strain KRS010.

## Figure S3


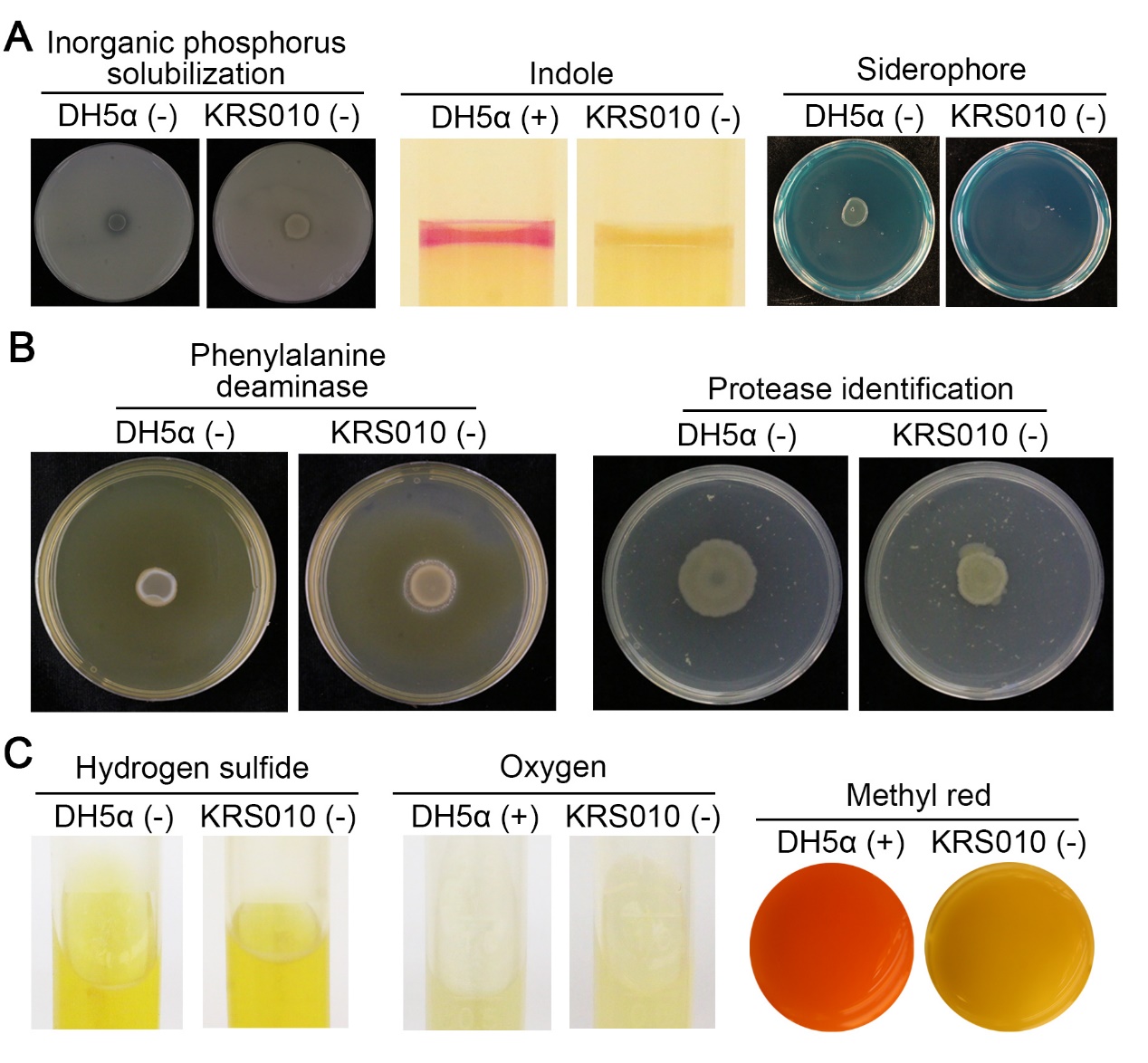


**Figure S3. Analysis of physiological and biochemical characteristics in *B. altitudinis* KRS010. (A)** Determination of relevant indicators for promoting growth. **(B)** Enzyme assay of strain KRS010. *E. coli* DH5a was severed as the negative control. **(C)** Determination of other physiological and biochemical indicators of strain KRS010.

## Figure S4


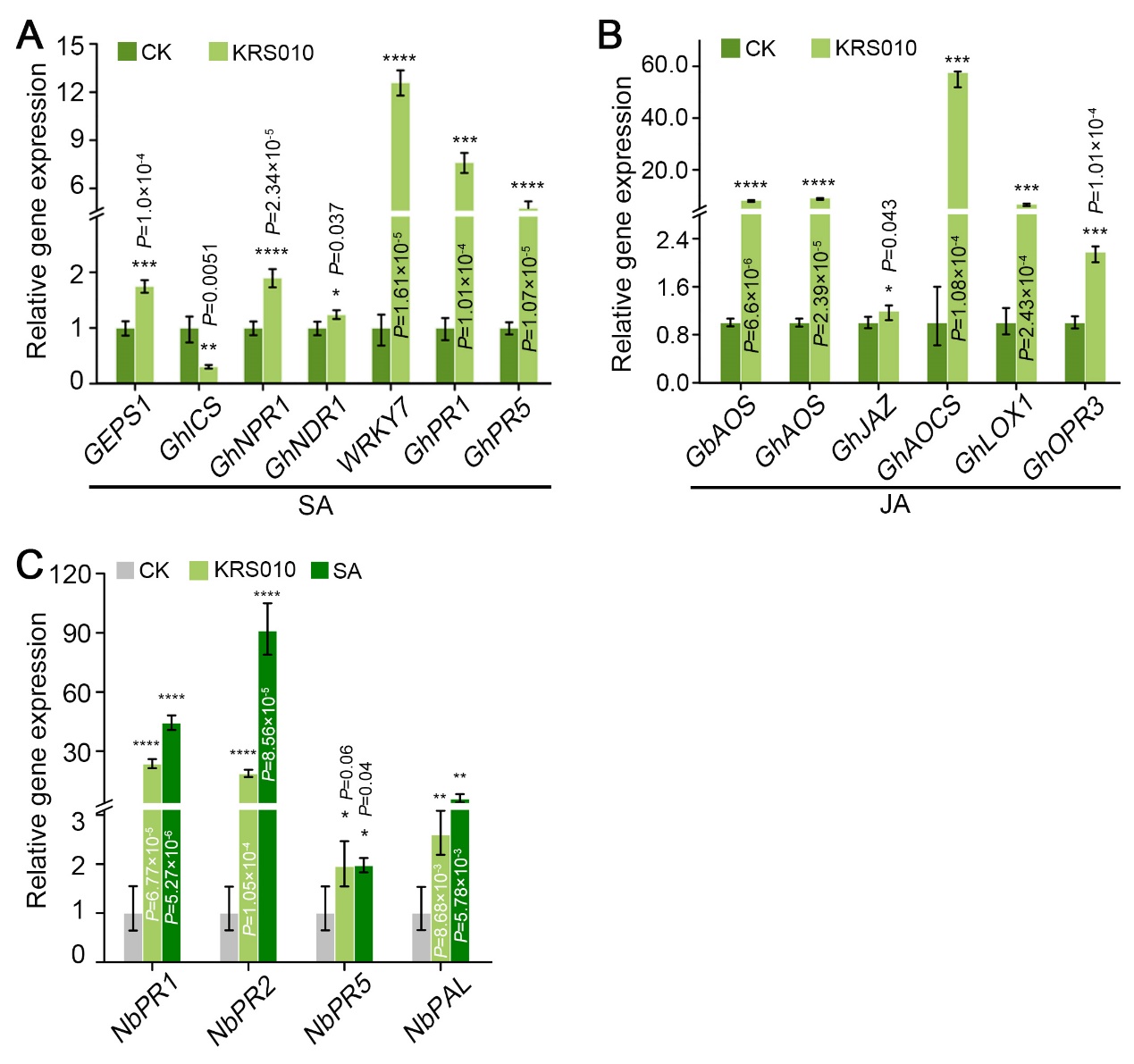


**Figure S4. Relative expression of marker genes in plant treated with KRS010 detected by RT-qPCR. (A, B)** After irrigating treatment, the related genes expression levels of cotton roots salicylic acid (SA) and jasmonic acid (JA) signaling pathway were examined, respectively. **(C)** The related genes expression levels of *NahG* transgenic tobacco leaves SA signaling pathway were examined after spraying the solution of 10 μmol/L SA. Defense-related genes involved hypersensitivity response (HR). Salicylic acid (SA). Error bars represent standard errors. **, ***, and **** represent significant differences at *P* < 0.01, *P* < 0.001, and *P* < 0.0001, respectively, according to unpaired Student’s *t* test

## Figure S5


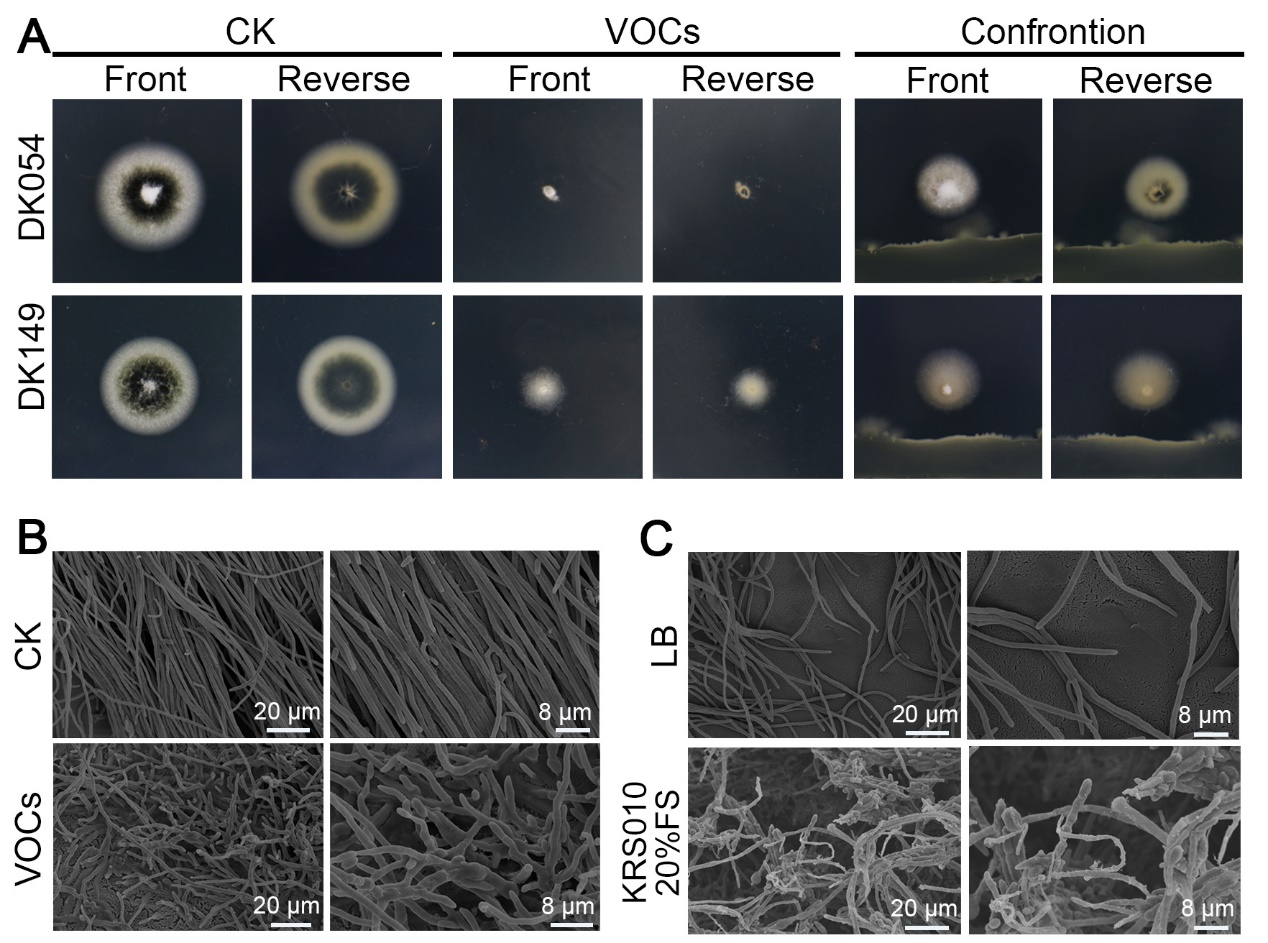


**Figure S5. KRS010 inhibits hyphal development and melanin production of *V. dahliae*. (A)** The growth phenotype of DK054 and DK149 was observed on PDA plates with confrontation culture and the covering fumigation of KRS010. **(B, C)** SEM was used to examine the hyphal morphology defects from the edges and superficial state of *V. dahliae* due to VOCs and the secondary metabolites of isolate KRS010, respectively.
